# Ear Acupressure, Heart Rate, and Heart Rate Variability in Patients with Insomnia

**DOI:** 10.1155/2013/763631

**Published:** 2013-02-11

**Authors:** Lu Wang, Weiping Cheng, Zhongren Sun, Yangyang Xu, Guangyu Cheng, Ingrid Gaischek, Haixue Kuang, Gerhard Litscher

**Affiliations:** ^1^Heilongjiang University of Chinese Medicine, Harbin 150040, China; ^2^Stronach Research Unit for Complementary and Integrative Laser Medicine, Research Unit of Biomedical Engineering in Anesthesia and Intensive Care Medicine, TCM Research Center Graz, Medical University of Graz, Auenbruggerplatz 29, 8036 Graz, Austria

## Abstract

This high-tech “teleacupuncture study” describes a neurovegetative ear acupressure effect in patients with chronic insomnia by using heart rate variability analysis. Heart rate (HR) and heart rate variability (HRV) measurements in 31 patients (mean age ± SD: 54.3 ± 10.6 years) were performed under standardized conditions in Harbin, China, and the data analysis was performed in Graz, Austria. Similar to our previous clinical and basic teleacupuncture research works, the electrocardiograms (ECGs) were recorded by an HRV Medilog AR12 system during ear acupressure of the Shenmen point on the left ear. HR decreased significantly (*P* < 0.05) during and after acupressure stimulation. The effect was not visible after the first stimulation, rather it appeared in the phase following the second acupressure stimulation (10 min after the first stimulation). Total HRV showed significant stimulation-dependent increases (*P* < 0.05), immediately after each acupressure stimulation with a maximum after the third stimulation (20 min after the first stimulation), but there was no long-lasting effect. The present results can serve as a solid basis for the further investigations of auricular point stimulation for noninvasive complementary use in treating insomnia.

## 1. Introduction

The term “teleacupuncture” was first mentioned worldwide by our research group of the Medical University of Graz, Austria, Europe, at the International Symposium “Modernization of Traditional Chinese Medicine” in May 2009. Today (December 21, 2012) a google search for “tele-acupuncture” yields 650,000 results.

This high-tech clinical and basic acupressure study using a “transcontinental teleacupuncture” design deals with the acute effects of ear acupressure on heart rate (HR) and heart rate variability (HRV) in patients with chronic insomnia. HR and HRV have been applied to understand autonomic changes during sleep and insomnia. One definition of insomnia is “difficulties initiating and/or maintaining sleep, or nonrestorative sleep, associated with impairments of daytime functioning or marked distress for more than 1 month” [1]. It has become a global health problem. The aim of this study was to test the hypothesis that patients with insomnia will demonstrate acute neurovegetative effects such as decreased HR and increased HRV during and after ear acupressure treatment as measured by continuous electrocardiographic monitoring and spectral analysis techniques.

In the scientific database PubMed (http://www.pubmed.gov/), there are more than 2,400 reviews on this topic. A research group from Taiwan published a randomized controlled trial in 2010 which showed effectiveness of acupressure for residents of long-term care facilities with insomnia [2]. 

## 2. Materials and Methods

### 2.1. Patients

In total, 31 patients (6 male, 25 female) with a mean age of 54.3 ± 10.6 (SD) years (range: 39–82) were investigated in this transcontinental clinical and basic research study. They all presented themselves at the hospital due to chronic insomnia. The Athens Insomnia Scale (AIS) was used for classification of the disease [3] (inclusion criteria: AIS ≥ 7). The scores ranged from 7 to 22, resulting in a mean value of 14.7 ± 4.4 (SD). The subjects had no obvious history of heart disease, cerebrovascular disease, or respiratory or neurological problems. The patients were fully informed about the nature of the investigation, and they all provided their informed consent. The methodological procedure including the registration of the noninvasive parameters was approved by the local ethics committee and was in accordance with the Declaration of Helsinki of the World Medical Association.

### 2.2. Electrocardiographic Monitoring

An HRV Medilog AR12 (Huntleigh Healthcare, Cardiff, UK, and Leupamed GmbH, Graz, Austria) system was used for bioelectrical cardiographic (ECG) recording. The data were analyzed using the “Fire of Life” software (Huntleigh Healthcare, Cardiff, UK) [4, 5]. The sampling rate of the recorder is 4096 Hz, allowing R waves to be detected extremely accurately. All raw data are stored on a compact flash memory card. The data are then read by an appropriate card reader connected to a standard computer and sent to the Stronach Research Unit at the Medical University in Graz. Biosignal ECG registration was performed in Harbin with three adhesive electrodes (Skintact Premier F-55; Leonhard Lang GmbH, Innsbruck, Austria) applied to three standard positions on the chest.

HR and HRV, which is the percentage change in sequential chamber complexes called RR intervals, can be calculated from the ECG. HRV can be quantified in the time and frequency domains using ECG power spectra [5–7]. These parameters are recommended by the Task Force of the European Society of Cardiology and the North American Society of Pacing and Electrophysiology [8]. Similar to previous studies of our research team, the mean HR, total HRV, LF (low frequency) and HF (high frequency) bands, and the LF/HF ratio of the HRV were evaluated [9–11].

### 2.3. Ear Acupressure Stimulation and Procedure

**Figure 1:**
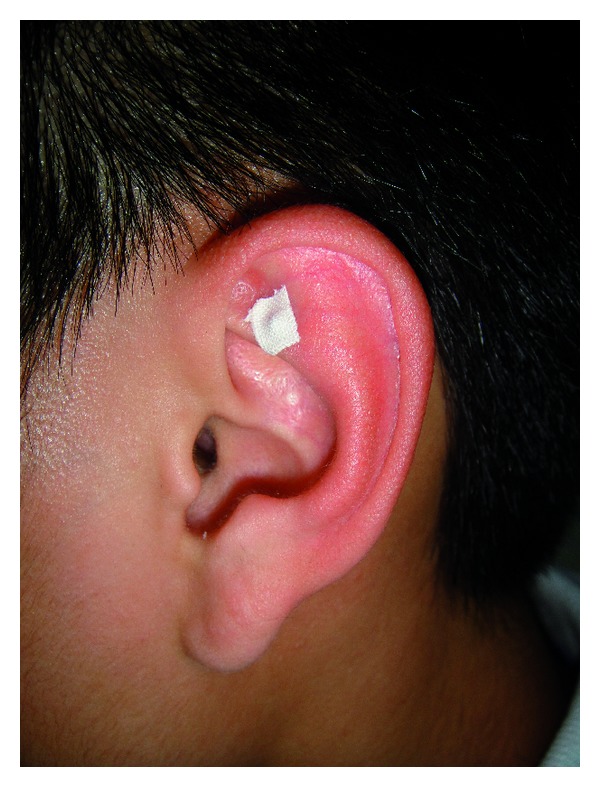
Location of the auricular acupoint Shenmen.

Auricular acupoint Shenmen on the left ear was selected for acupressure stimulation. The ear point Shenmen is located at the lateral third of the triangular fossa, in the bifurcating point between superior and inferior crura of antihelix (see Figure 1). This point is indicated mainly for insomnia, pain, and skin itching. Its action is thought to ease the mind, relieve pain, and subdue inflammation [12].

Semen vaccariae (Wang Bu Liu Xing, cowherb seed; globes of ~1.5 mm in diameter; surface: smooth; color: black; Hebei, China) was applied unilaterally onto the acupoint Shenmen (TF 4) of the left ear by adhesive plaster. 

The auricular acupressure was performed for 15 seconds each time, with two pressure movements per second, resulting in a total of 30 pressure movements per stimulation. Acupressure was applied every 10 minutes, for a total of 3 times during the entire measurement period of each patient.

**Figure 2:**
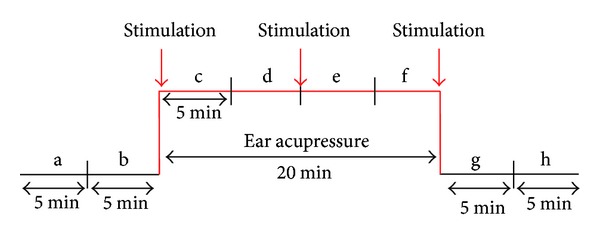
Experimental protocol for auricular acupressure at the Shenmen ear acupuncture point.

The measurement profile and measurement times (a–h) are shown schematically in Figure 2. Eight measurement periods were compared: two phases before stimulation (a, b), four phases during which acupressure stimulation was performed (c–f), and two phases after acupressure (g, h).

### 2.4. Statistical Analysis

The data were analyzed using Friedman repeated measures analysis of variance (ANOVA) on ranks (SigmaPlot 12.0, Systat Software Inc., Chicago, USA). Post hoc analysis was performed using the Tukey test. The level of significance was defined as *P* < 0.05.

## 3. Results

**Figure 3:**
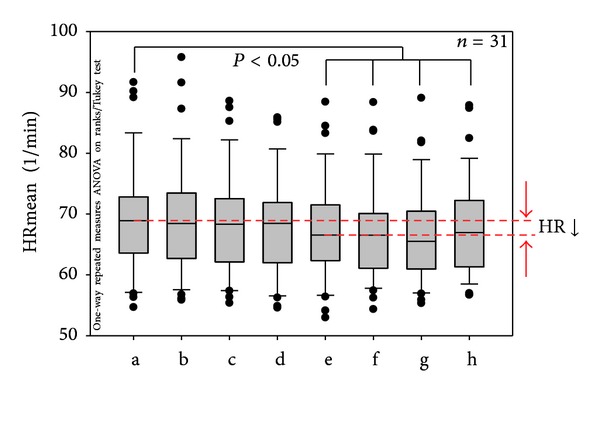
Box plots displaying the mean heart rate (HR) of the 31 patients. Note the significant decrease beginning in phase (e). The ends of the boxes define the 25th and 75th percentiles with a line at the median and error bars defining the 10th and 90th percentiles. The different measurement phases (a–h; cf. Figure 2) are indicated.

Mean HR is shown in Figure 3. In this figure, the results from 31 patients for measurement phases a–h (before, during, and after stimulation of the Shenmen auricular acupoint) are documented. There was a significant (*P* < 0.05) decrease in HR, starting after the second acupressure stimulation (phase e) and lasting until the end of the recording period compared to the first control interval (a) before stimulation. After the third acupressure stimulation, the HR reached its minimum (phase g). In the last phase (h), five minutes after the last stimulation, HR increased again slightly; however, it was still significantly reduced compared to the baseline value before stimulation (phase a).

**Figure 4:**
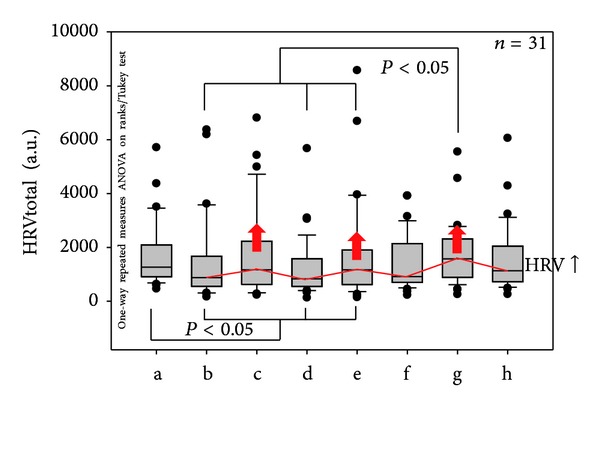
Statistical analysis and box plot illustration of HRVtotal of the 31 patients with insomnia. Note the stimulation-dependent increases in HRVtotal (red arrows). For further explanations, see Figure 3.

Figure 4 shows stimulation-dependent increases of total heart rate variability (HRVtotal). The maximum increase of HRVtotal occurred after the third acupressure stimulation at the ear. This increase was not only significant compared to the control phase before stimulation, but also compared to the phase after the second acupressure stimulation (*P* < 0.05).

The analysis of the LF/HF ratio did not show any significant differences between the phases before, during, and after acupressure stimulation.

## 4. Discussion

Acupressure is one of the most commonly used treatment methods in China. It is especially used for very young and old patients. Persons can use it by themselves, or assisted by family members, at home. There are courses in China on TV or in newspapers on how to perform acupressure. There is still a need for scientific investigations concerning acupressure especially ear acupressure. In the scientific database PubMed (http://www.pubmed.gov/), one can find about 30 times more articles on acupuncture than on acupressure (December 21, 2012). There are more clinical reports on acupressure than basic research on this topic. Up to now, there are no standard stimulation parameters which are easy to reproduce and/or compare; one only needs to mention the subject-dependent pressure applied on the skin. Usually, acupressure is performed manually, but there are also some innovative approaches and developments to apply vibration or acupressure stimulation via electronic devices. First results concerning this high-tech acupressure stimulation have been presented by our group in the field of ear acupressure [13].

As another treatment modality of traditional Chinese medicine, auricular acupressure is characterized by easy manipulation, sustained local stimulation, a wide range of appropriate indications, and cost efficiency with effective results. With its many benefits, ear acupressure has the potential to become one of the most commonly applied treatments intending to promote relaxation, relieve pain and discomfort in the body, and treat insomnia.

Various studies have shown that acupressure is effective for treating insomnia. In 2005, a research group from the College of Nursing Science at Kyung Hee University in Korea designed a triangulation study to observe the effects of auricular acupuncture on insomnia in Korean elderly [14]. Another group in Taiwan conducted a study in 2011 which found the relationship of subjective sleep quality and cardiac autonomic nervous system in postmenopausal women with insomnia under auricular acupressure [15]. 

Single-point treatment, which has been more frequently used in acupuncture investigations nowadays, might be important to prove the point specificity by using different means of acupoint stimulation. 

We have already investigated the effects of Shenmen (H7) on the left hand on HRV in patients with insomnia in one of our previous teleacupuncture studies between Harbin and Graz [11]. In order to compare the complementary effects and also the specificity of different single acupoints in the treatment of insomnia patients, ear acupressure on Shenmen on the left ear is conducted in this study on chronic insomnia in adults of different ages. 

Similar results have been obtained in our recently published Sino-European high-tech acupuncture study. By using single-point acupuncture, this study has shown decreased HR and increased HRV acute effects on insomnia patients during and after acupuncture treatment [11].

**Our Study Has Some Limitations**

First, there is no clinical outcome data of the treatment's effect on patients' insomnia correlating with the data of HR and HRV. Secondly, there is no control group in this study. Without a sham point control that has no known effect on insomnia, it is not clear whether our results were due to a specific effect of the classically used acupuncture point for insomnia, or merely due to a nonspecific effect. In future investigations, we will use another ear point (e.g., ear point tonsil, *biantaoti*) as a sham point.

## 5. Conclusions

The following conclusions can be drawn from the results of the present first transcontinental teleacupressure study in patients with insomnia.

- Heart rate decreased significantly during and after acupressure stimulation of the Shenmen acupuncture point on the left ear. The effect was not visible after the first stimulation, rather it appeared in the phase following the second acupressure stimulation (10 min after the first stimulation).
- Total HRV showed significant stimulation-dependent increases, immediately after each acupressure stimulation with a maximum after the third stimulation (20 min after the first stimulation), but there was no long-lasting effect.
